# Successful rituximab treatment of granulomatosis with polyangiitis with cranial neuropathies

**DOI:** 10.1186/s41232-018-0079-4

**Published:** 2018-11-19

**Authors:** Maho Nakazawa, Katsuya Suzuki, Hidekata Yasuoka, Kunihiro Yamaoka, Tsutomu Takeuchi

**Affiliations:** Division of Rheumatology, Department of Internal Medicine, Keio University School of Medicine, 35 Shinanomachi, Shinjuku-ku, Tokyo, Japan

**Keywords:** Granulomatosis with polyangiitis, Cranial neuropathies, Rituximab, Drug resistance, B-lymphocytes

## Abstract

**Background:**

In granulomatosis with polyangiitis (GPA), peripheral nerve involvement is common but central nervous system (CNS) involvement is extremely rare and treatment strategy has not been established. We report a case of intravenous cyclophosphamide (IVCY)-resistant GPA with associated cranial neuropathies that was successfully treated with rituximab (RTX).

**Case presentation:**

A 37-year-old man with intractable sinusitis had several months of headache, hoarseness, and dysphagia; a month of right-sided deafness and nasal bleeding; and a week of dysarthria, steppage gait, and numbness in the right L5 distribution. A magnetic resonance imaging (MRI) examination of the head showed an infiltrative lesion in the right skull base encasing the carotid sheath. Computed tomography (CT) scan of the chest revealed a 23 mm nodule in the left upper lobe. Histology was inconclusive. Therefore, the patient was diagnosed as GPA. He was treated with glucocorticoids (GC) and IVCY. Three months later, he was readmitted for recurrence of headache and new left-sided hearing loss. He was treated with GC and RTX, and a 1-year remission followed. The molecular mechanism of RTX is not fully understood. In this case, RTX was more effective at rapidly and strongly suppressing B cells than CY. Since the B cell count was proportional to the patient’s clinical manifestations, B cells might represent a suitable target for the treatment of GPA with cranial neuropathies.

**Conclusions:**

GPA with cranial neuropathies might be useful with RTX as induction therapy.

## Background

Granulomatosis with polyangiitis (GPA) is a systemic necrotizing granulomatous vasculitis that involves the upper airways, lungs, and kidneys. While peripheral nerve involvement is common, central nervous system (CNS) involvement is extremely rare [1, 2]. We report the successful treatment of a case of cyclophosphamide (CY)-resistant GPA with cranial neuropathies with rituximab.

## Case presentation

A 37-year-old man was admitted to hospital for several months of headache, hoarseness and dysphagia; a month of right-sided deafness and nasal bleeding; and a week of dysarthria. He had experienced sinusitis for 1 year before admission and had been treated with antibiotics. He was successfully treated with glucocorticoids (GC) for sudden right-sided hearing loss 9 months before admission. His body weight had decreased by 10 kg over the previous month. A week before admission, he developed a right steppage gait and numbness in the right L5 distribution.

**Fig. 1:**
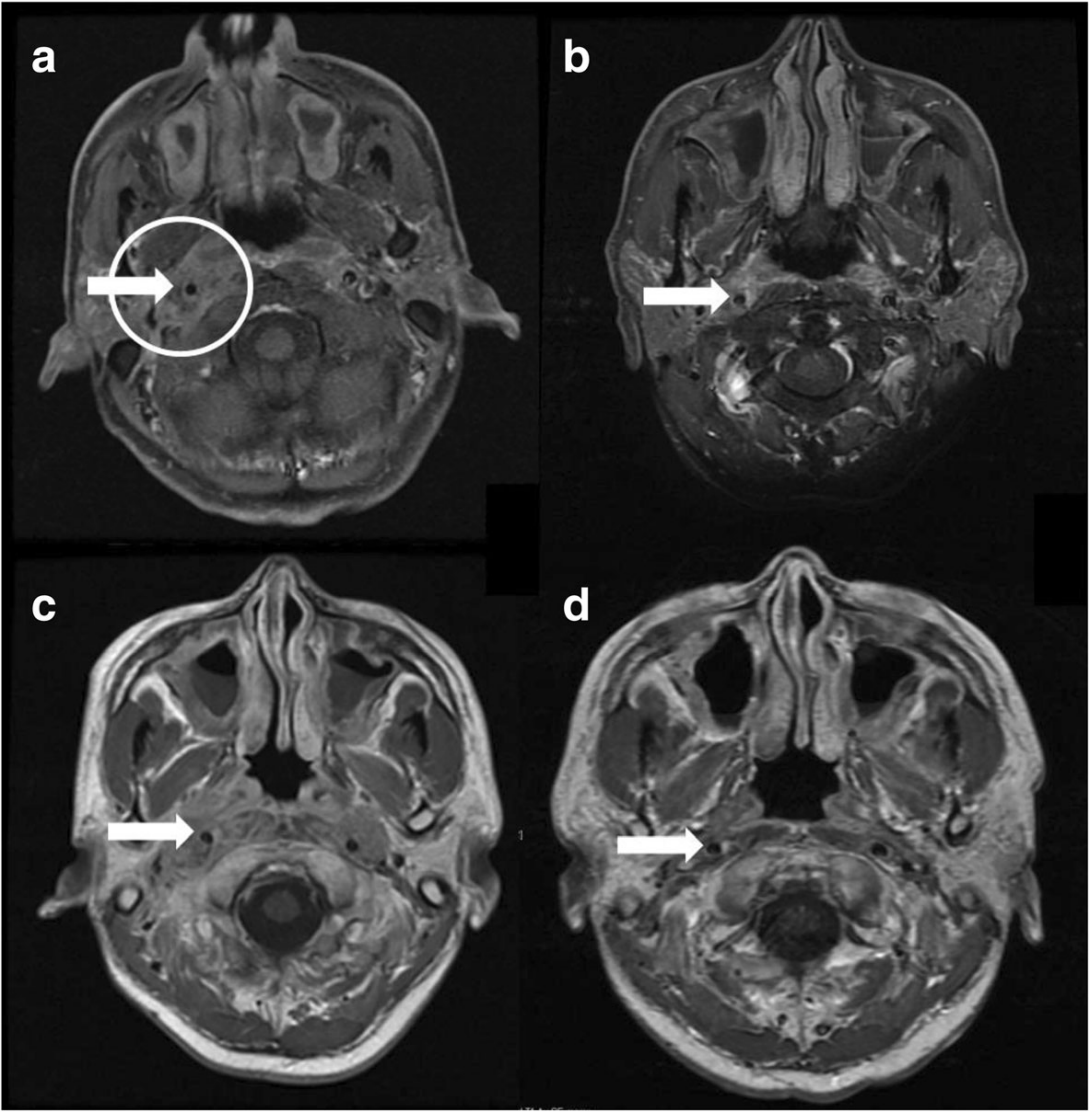
Magnetic Resonance (MR) imaging of the head. Fat-suppressed T1-weighted gadolinium-enhanced axial scans through the skull base (**a**, **b**), and T1-weighted gadolinium-enhanced axial scans of the skull base (**c, d**). There is bilateral maxillary sinusitis and an infiltrative lesion of the right retropharynx (circle) around the internal carotid artery (arrow). Images were taken **a** on admission, **b** after the 1st IVCY, **c** before RTX treatment, and (**d**) 6 months after RTX treatment. (**d**) The sinusitis and the infiltrative lesion were markedly improved after RTX treatment

On admission, body temperature was 37.7 °C and the rest of his vital signs were normal. Neurological examination showed a bilateral mixed hearing loss, a right curtain sign, weakness of the right trapezius, rightward tongue deviation, and paralysis of the right peroneal nerve. Initial blood tests showed a slightly elevated erythrocyte sedimentation rate (29 mm/h) and C-reactive protein (CRP) levels (1.06 mg/dL), and white blood cell count was slightly increased (8.9 × 109/L). His renal and liver function was normal (eGFR 118 ml/minute/1.73 m2) and the urine test was also normal (proteinuria, hematuria, urinary cast were negative). Anti-nuclear antibody, rheumatoid factor, angiotensin converting enzyme, myeloperoxidase-anti-neutrophil cytoplasmic antibody and soluble interleukin-2 receptor were normal, but proteinase 3-anti-neutrophil cytoplasmic antibody was increased (16.9 IU/mL). Cerebrospinal fluid was normal. A gadolinium-enhanced MRI scan of the head showed an enhancing infiltrative lesion in the right retropharynx encasing the carotid sheath (Fig. 1a), which seemed to cause the paralysis of IX, X, XI and XII nerves. Lumber spine MRI showed no evidence of lumbar disk herniation and nerve conduction study showed the paralysis of the right peroneal nerve. Chest computed tomography showed a 23 mm nodule in the left upper lobe. A CT-guided needle biopsy of the lung lesion and biopsy of the nasal mucosa were performed but showed only infiltration of inflammatory cells and no evidence of malignancy, vasculitis or granuloma. Culture of the lung specimen showed no evidence of infection and the interferon-gamma release assay for Mycobacterium tuberculosis was negative. We diagnosed GPA based on the American College of Rheumatology classification criteria for Wegener’s granulomatosis (WG) and classification of WG by the Watts algorism [3, 4].

**Fig. 2:**
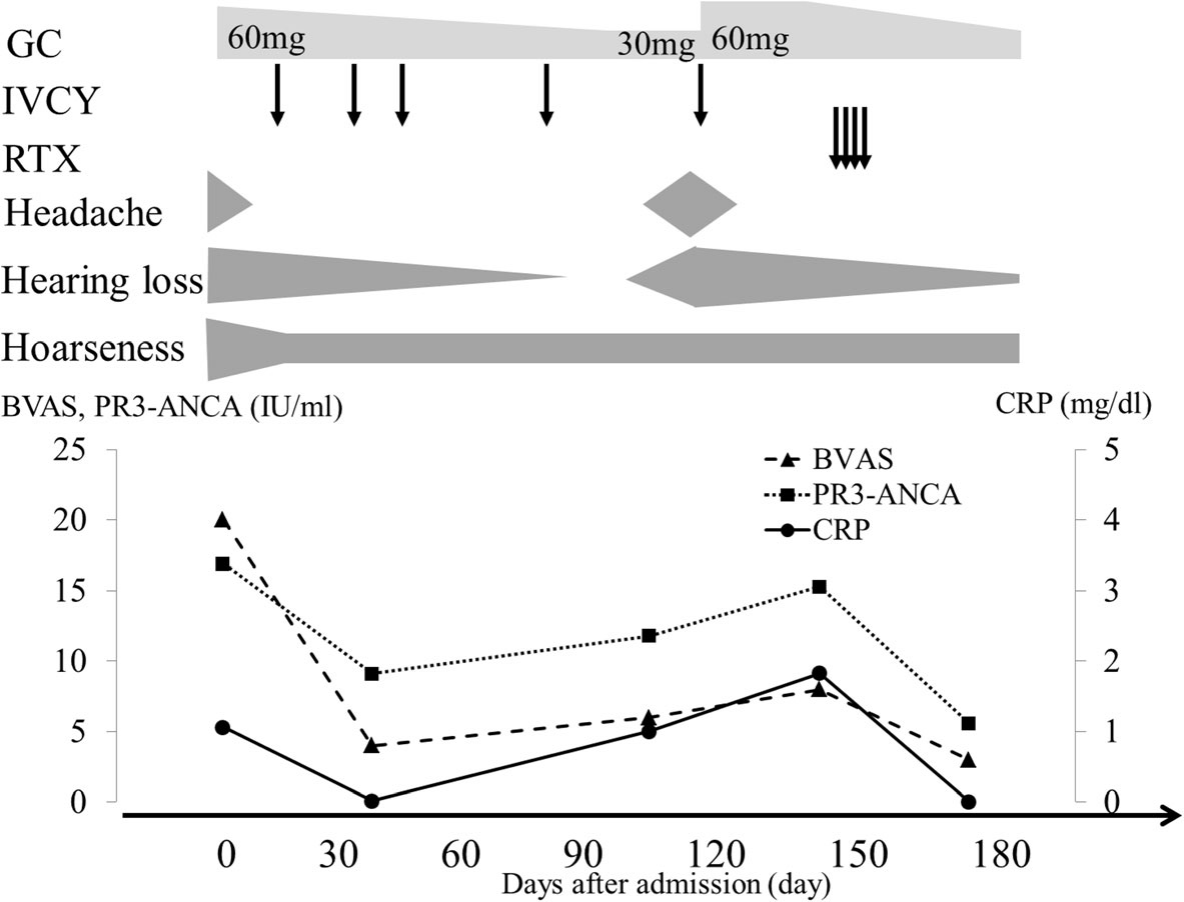
Clinical course. IVCY dose and interval were 1000 mg every 3 weeks and RTX dose and interval were 600 mg every week. GC, glucocorticoid; IVCY, intravenous cyclophosphamide; RTX, rituximab; BVAS, Birmingham Vasculitis Activity Score 2008 version 3; PR3-ANCA, proteinase 3-anti-neutrophil cytoplasmic antibody; CRP, C-reactive protein

The patient was treated with prednisone (PSL) 1 mg/kg daily and IVCY 1000 mg every 3 weeks (Fig. 2). His fever, headache, and swallowing function improved rapidly, and peripheral neuropathy also improved, but hoarseness persisted. His Birmingham Vasculitis Activity Score 2008 version 3 score improved from 14 to 4, and he was discharged on IVCY and a tapering dose of PSL 45 mg/day [5].

Three months later, while receiving oral GC and IVCY, he was readmitted for recurrence of headache and right-sided hearing loss. On MRI, the lesion encasing the right carotid sheath has not reduced (Fig. 1b). CRP levels remained slightly increased (1.04 mg/dL). He was treated with RTX 600 mg/week for 4 weeks and GC for re-induction therapy. His headache improved rapidly and hearing ability improved slowly. He was still on GC 1 year after RTX administration, with persistent hoarseness as his only symptom.

**Fig. 3:**
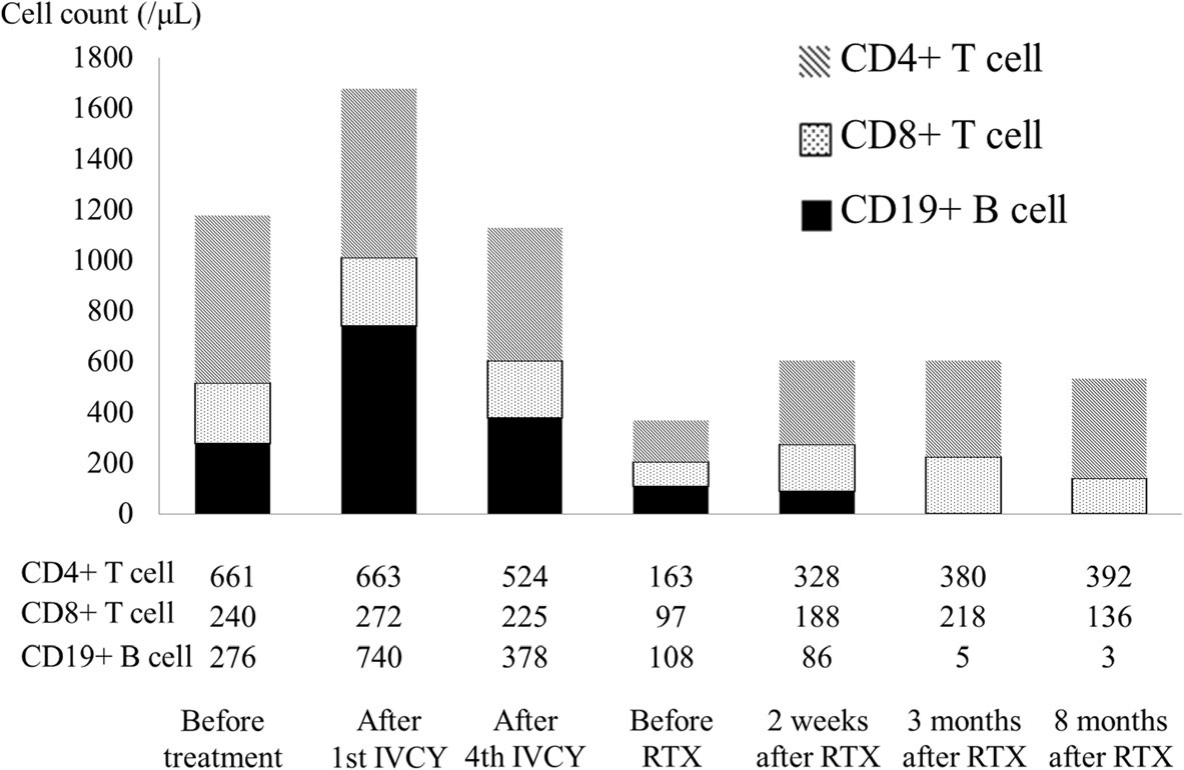
Changes in peripheral lymphocyte count. CD4, CD4+ T cell; CD8, CD8+ T cell; CD19, CD19+ B cell

To investigate immune status, we tracked peripheral T and B cell counts after RTX administration (Fig. 3). According to the peripheral T and B cell counts, whereas CY treatment with GC reduced the number of peripheral CD4+ and CD8+ T cells and B cells, RTX selectively reduced the number of peripheral B cells slowly over time. B cells tended to decrease 2 weeks after RTX treatment, with the reduction lasting 8 months.

## Discussion and conclusions

We encountered a case of GPA with lower cranial and peripheral neuropathies. Although GPA often displays neurological involvements, they are mostly peripheral neuropathies and cranial neuropathies are extremely rare. Among cranial neuropathies, optic nerve neuropathy is the most common [6]. The pathogenesis of cranial neuropathies is still unclear because biopsy of the lesion is difficult and risky. It is unclear whether cranial neuropathies arise due to mechanisms of vasculitis or granuloma formation.

We used the PubMed database to retrieve all articles written in English containing the descriptors GPA and cranial neuropathy in their titles or texts, including treatment regimen. The nine known cases of GPA with cranial nerve involvement are listed according to treatment in Table 1 [6–13]. Similar to our case, most cases with lower cranial neuropathies had MRI findings of a unilateral skull base process encasing the carotid sheath. The inflammation of upper respiratory tract lesion might progress posteriorly and extend into the retropharyngeal space. Recently, RTX was shown to be effective for treating anti-neutrophil cytoplasmic antibody-positive GPA and microscopic polyangitits. (RAVE study) [14]. However, the effectiveness of RTX in CNS involvement was not evident in the study. Most previous case reports have treated patients with CY for induction therapy, with few receiving GC monotherapy or methotrexate. One patient who was treated with CY had a recurrence and then RTX for re-induction therapy was effective, but long term effectiveness was not shown [6]. The present case is the second case to show the efficacy of RTX in refractory GPA with cranial neuropathies, which induces remission for 1 year.

**Table 1:** Reported cases of GPA with cranial neuropathies

Reference	Age and sex	Signs and symptoms	Cranial nerve involvement	MRI findings	Treatment for induction	Outcome
[7]	23F	Otalgia, facial paralysis, dysphagia	V, IX, X	Mass on right skull base encasing the carotid sheath	GC + CY	No recurrence for 1 year
[8]	42F	Dysphagia, paresis of fifth nerve, genioglossus, trapezius, and sternocleidomastoid	V, IX, X, XI, XII	Normal	GC + oral CY	No recurrence for 30 months
[6]	73F	Dysarthria, left hearing loss, paresis of hypoglossal nerve	VIII, IX, X, XII	Mass on left skull base encasing the internal carotid artery	GC + intravenous CY	Failure for re-induction
[9]	35 M	Dysphagia, paresis of vagus and accessory nerve	X, XI	Not performed (brain CT was normal)	GC + CY	No recurrence for 6 months
[10]	30F	Dysarthria, paresis of hypoglossal nerve	XII	Right-sided retropharyngeal mass effacing the carotid sheath	GC + MTX	Not described
[11]	42 M	Hearing loss, facial nerve palsy	VII, VIII	Not described	GC + oral CY	Failure for re-induction
[12]	69 M	Diplopia	VI	Normal	GC + oral CY	No recurrence for 4 months
[12]	31F	Hoarseness, dysphagia, hypoglossal nerve and abducens nerve palsies	VI, X, XI, XII	Normal	GC + CY	Not described
[13]	56F	Facial palsy and hearing loss	VII, VIII	Mass on central and posterior skull base adjoinin the clivus and jugular foramen	GC	Not described

*GC* glucocorticoid, *MTX* methotrexate; *CY* cyclophosphamide

Another study that demonstrated the efficacy of RTX in refractory GPA showed complete remission/improvement in 89.2% of patients with renal disease and 80.8% with alveolar hemorrhage, but only 44.4% of those with orbital masses and 49.9% with pachymeningitis [15], suggesting that RTX may be more effective for vasculitic than granulomatous manifestations. While involvement of granulomatous regions was suspected in this cranial neuropathy case [7], RTX was successfully effective. Further case series study would be needed.

The molecular mechanism of RTX is not fully understood. We speculated that RTX was more effective at rapidly and completely suppressing B cells than CY (Fig. 3). Since B cell count was proportional to the clinical manifestations, B cells might represent a good target for the treatment of GPA with cranial neuropathies. In these cases, rapid suppression of B cells by RTX might be critical.

In conclusions, GPA with cranial neuropathies might be effective with RTX as induction therapy.

## Abbreviations

CNS: central nervous system
CRP: C-reactive protein
CT: computed tomography
GC: glucocorticoids
GPA: granulomatosis with polyangiitis
IVCY: intravenous cyclophosphamide
IVCY: intravenous cyclophosphamide
MRI: magnetic resonance imaging
PSL: prednisone
RTX: rituximab
WG: Wegener’s granulomatosis
